# Emotion regulation-enhanced group treatment for gambling disorder: a non-randomized pilot trial

**DOI:** 10.1186/s12888-021-03630-3

**Published:** 2022-01-06

**Authors:** Viktor Månsson, Olof Molander, Per Carlbring, Ingvar Rosendahl, Anne H. Berman

**Affiliations:** 1 Center for Psychiatry Research, Department of Clinical Neuroscience, Karolinska Institutet, & Stockholm Health Care Services, Region Stockholm, Norra Stationsgatan 69, 7tr, SE-113 64 Stockholm, Sweden; 2 Department of Psychology, Stockholm University, Stockholm, Sweden; 3 Department of Psychology, Uppsala University, Uppsala, Sweden

**Keywords:** Gambling disorder, Cognitive behavioral therapy, Emotion regulation, Pilot trial, Mixed methods

## Abstract

**Background:**

Despite the association of Gambling Disorder (GD) with poor mental health, treatment options generally lack components targeting emotional difficulties. This study investigated the feasibility and acceptability of adding strategies of emotion regulation to an eight-session weekly group treatment.

**Method:**

This non-randomized pilot study recruited 21 treatment-seeking adults with GD, (mean age = 36.3, 19% females) from addiction care. In a mixed methods design, measures of within-group changes in self-reported symptoms of GD were complemented with thematic analysis of post-treatment interviews regarding the feasibility of the treatment.

**Results:**

Within-group scores on the Gambling Symptoms Assessment Scale (G-SAS) showed a 47% decrease (β: -0.1599, 95% CI: − 0.2526 to − 0.0500) from pre-treatment to 12-month follow-up, with Hedges’ *g* = 1.07 (CI: 0.57–1.60).

The number of GD-symptoms according to the Structured Clinical Interview for Gambling Disorder (SCI-GD) decreased from 7.0 (SD = 1.60) at pre-treatment to 2.1 (SD = 2.36) at 12-month follow-up. Participants completed an average of 6.3 sessions and rated the intervention high in satisfaction and acceptability. Feasibility interviews showed no noticeable negative effects or ethical issues. Furthermore, helpful components in the treatment were: increased awareness of emotional processes and strategies to deal with difficult emotions.

**Conclusions:**

Adding emotion regulation strategies in the treatment of GD is feasible and acceptable and warrants further investigation in a controlled trial.

**Trial registration:**

This study was registered with ClinicalTrials.gov (Identifier NCT03725735).

## Background

Gambling Disorder (GD) has a severe negative impact on individuals’ mental health and presents a societal challenge. The term problem gambling, encompassing sub-clinical symptoms as well as GD, is commonly employed in prevalence studies to refer to a continuum of gambling behavior that leads to negative consequences for individuals, their families and/or other significant others. Population surveys worldwide vary in their estimates of problem gambling, indicating that between 0.2 and 5.3% of the adult population suffer from problem gambling the past 12 months [1]. In addition, suicide rates are elevated and individuals with GD usually show low service utilization, due to shame, denial and not wanting peers to find out about their gambling problems [2–4]. In Sweden, although gambling is regulated by the state and gambling participation has declined in recent years, the proportion of problem gambling seems relatively stable over the past two decades [5, 6]. In parallel, circumstances around the gambling market have changed markedly with the emergence of online casinos, leading to increased availability of games offering quick return and continuous play. Among those seeking treatment for GD in Sweden, online casinos and online sports betting are the games most commonly reported [7].

There is support for the efficacy of Cognitive Behavioral Therapy (CBT) in reducing time and money spent on gambling and problem gambling behaviors, at least in the short term, as a Cochrane systematic review of the psychological treatment for problem gambling has shown [8]. However, treatment research has been characterized by a large variation in outcome measures [9], high dropout rates and a scarcity of follow-ups exceeding 3 months [10]. A potential complicating factor in designing and delivering treatment is the mental health burden among individuals with GD, which has been recognized in a number of studies, both among treatment-seekers and in community samples. Research indicates that disorders most commonly occurring with GD include affective disorders (23.1–37.9%) and anxiety disorders (17.6–37.4%) [11, 12]. Among treatment-seekers with GD in Sweden, 58% were assessed as having at least one additional psychiatric disorder [7] and nationwide registry data from specialized health care units have shown registered comorbid diagnoses at an even higher rate of 73% [13], confirming the heterogeneity and the mental health burden among this group.

The identification of multiple mental health problems in an individual may reflect a symptomatic, transdiagnostic expression of the individual’s overall strategies when coping with emotional experiences or strong impulses. One such strategy concerns *emotion regulation* (ER), where several studies have shown correlations between problem gambling and difficulties in ER [14–19]. Explicit strategies of dealing with difficult emotions have been conceptualized as utilizing *reappraisal*, i.e., changing the interpretation of a self-relevant meaning of a stimulus in order to reduce or alter its emotional impact or *control or suppression* of one’s behavior in order to decrease expressive behavior, but not necessarily the emotional experience [20]. From a brain-behavior perspective, these explicit strategies have been shown to include activation of various brain regions, such as the dorsolateral prefrontal cortex, the ventrolateral prefrontal cortex, the parietal insula, the insula and the parietal cortex, and the supplemental motor area. Implicit ER-strategies, on the other hand, are processed automatically, without conscious surveillance, and are associated with activation of the ventral anterior cingulate cortex and the ventromedial prefrontal cortex [21]. Difficulties at this automatic, implicit level have been hypothesized to interfere with loss-related learning processes in gambling [17], i.e., that repeated losses do not lead to a reduction of gambling. One argument for this is the finding that gambling can be reduced by simply providing feedback on actual losses [22, 23].

A more recent, modified definition describes ER as “adaptive ways of responding to emotional distress, including the awareness, understanding, and acceptance of emotions and an ability to control impulsive behaviors and engage in goal-directed behaviors when experiencing negative emotions” ([24], p. 2).

With this in mind, impaired impulse control and difficulties in engaging in goal directed behavior in the presence of emotional difficulties have both shown positive associations with having more severe gambling problems [15]. Accordingly, this is in line with the description that individuals with GD engage in gambling to moderate emotional difficulties and that deficits in strategies in regulating emotional experiences may play a key role in both the development and maintenance of gambling problems [25, 26].

Nonetheless, to our knowledge, no study has hitherto investigated the feasibility of including strategies of ER when treating individuals with GD. In view of the novelty of this approach, it is also important to gain insight into participants’ experiences of such treatment, their views on the feasibility and acceptability of the treatment, as well as quantitative outcomes over time. The objectives of this study are thus firstly to examine the acceptability and feasibility of the intervention and secondly to investigate any within-group changes on outcome measures of GD and psychiatric comorbidity, over a 12-month period following treatment.

## Methods

This non-randomized pilot study aimed to evaluate the feasibility and acceptability of ER-enhanced group CBT for individuals with GD and possible psychiatric comorbidity, using a mixed methods approach including quantitative outcome analyses over time and a thematic analysis of interviews on the feasibility, acceptability and potential negative treatment effects.

### Participants

In order to recruit a clinically representative sample, individuals from the waiting list at the Stockholm Dependency Center (SDC) were targeted for recruitment, between September 2017 and September 2018. Clinicians at the SDC informed prospective participants at intake interview and individuals awaiting group treatment for GD were informed through telephone about the study. In addition, letters with information about the study were also sent out to approximately 40 patients on the waiting list. At its peak, the waiting list included over 100 individuals; however, the majority were not actively seeking group treatment, but were awaiting individual treatment offers, or were non-responders to initial treatment offers. It is unknown exactly how many individuals received information about the study.

All potential participants filled out an online informed consent form and were also provided with oral information from the research group regarding the study procedures. After consenting, prospective participants were screened in a telephone interview by clinical psychologists for comorbid psychiatric disorders and symptoms of GD within the last 12 months. Demographic information, baseline data and weekly measures during treatment and follow ups were collected via an online survey tool [27]. Weekly measures were accessed through a weblink that was sent out the day before the group session. All available participants were interviewed post-treatment by an external interviewer, who was not known to the participants. The sampling for the qualitative interview was sequential [28], meaning participants who attended more than three sessions were invited to participate.

**Fig. 1:**
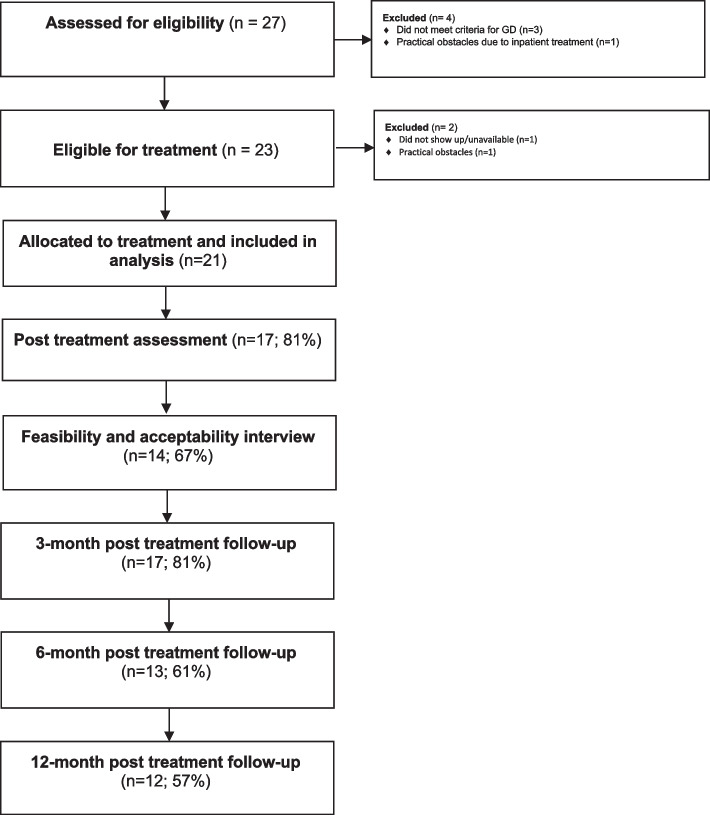
Participant flowchart

A total of 27 participants provided their informed consent and 21 were included in the study, with three-, six- and 12-month follow-ups dated from the post-treatment timepoint (see Fig. 1 for participant flow). Inclusion criteria consisted of 1) a diagnosis of GD according to the DSM-5; 2) being 18 years or older; 3) able to speak and read Swedish; and 4) available for participating in scheduled group sessions. Exclusion criteria were: 1) fulfilling criteria for manic episode and having reported gambling during such episodes (this is stated as an exclusion criterion in the DSM 5-diagnosis) and 2) acute psychiatric symptoms, where participants with identified ongoing psychotic symptoms or elevated risk of suicide were offered referral to psychiatric treatment. Table 1 shows participant characteristics.

**Table 1:** Participants’ demographic and clinical characteristics, *n* = 21

Age	Mean (SD)	36.3 (9.05)
	Range	25–57
Females	Frequency (%)	4 (19.0)
Occupation	Frequency (%)	
	Employed	13 (61)
	Sick leave	4 (14)
	Unemployed	2 (10)
	Student	1(5)
	Parental leave	1 (5)
Frequency of tentative comorbid diagnosis according to MINI-interview	Any depressive disorder	17 (80%)
	Panic Disorder	4 (19%)
	Alcohol Use Disorder	3 (14%)
	Bipolar Disorder	1 (5%)
	Social Anxiety	1 (5%)
	Substance Use Disorder	1 (5%)
	Antisocial Personality Disorder	1 (4%)
	Psychotic symptoms	1 (4%)
	PTSD	1 (4%)
Duration of illness (months)	Mean	
		88
Type of games played	N (%)	
	Online casino	11(52%)
	Online sports betting	6 (29%)
	Electronic Gaming Machines	5 (24%)
	Poker online	5 (24%)
	Land-based poker	4 (19%)
	Bingo/lottery	4 (19%)
	Casino	3 (14%)
	Horse betting	3 (14%)
	Stock market	1 (5%)

### Diagnostic assessment

The Mini International Neuropsychiatric Interview, version 7 (MINI-7 [29];) was used as a screener for psychiatric diagnoses. Symptoms of GD according to the Diagnostic and Statistical Manual of Mental Disorders (DSM- 5 [30];) were assessed using the Structured Clinical Interview for Gambling Disorder (SCI-GD) [31], updated from the previous SCI-Pathological Gambling to harmonize with the DSM 5 diagnosis. For the purpose of this study participants were asked to describe lifetime occurrence of a period when gambling was most frequent and intense, as well as the occurrence of symptoms of GD within the last 12 months. Each participant received a score between 0 and 9 (diagnostic threshold of 4) for symptoms within the last 12 months. The SCI-GD was administered at baseline and 12 months post-treatment.

### Quantitative measures

The primary outcome was symptoms of GD according to the Gambling Symptoms Assessment Scale (G-SAS [32];), a 12-item scale assessing symptoms of GD within the last week, such as thoughts, urges and anticipatory experiences related to gambling and gambling urges. For example, item 1 asks: *“If you had unwanted urges to gamble during the past week, on average, how strong were your urges?”*. Each item was scored between 0 and 4 with a maximum total score of 48. Higher scores indicated more severe symptoms. The G-SAS has shown good internal consistency (α = .89) and has been found to be reliable and valid in assessing changes in symptoms during a treatment study [32]. Internal consistency in the present sample was excellent (α = .90). Suggested cutoff scores for levels of severity are: > 40 equivalent to extremely severe symptoms, 31–40 severe symptoms, 21–30 moderate symptoms and 8–20 mild symptoms and < 7 minimal symptoms An additional primary outcome - money and time spent gambling - was measured on a weekly basis during treatment by asking: (a) how much money have you spent gambling during the past week?, and (b) how much time have you spent gambling during the past week?

Additional outcomes, consisting of symptoms of depression and anxiety, were administered prior to treatment, as weekly measures during the 8 weeks of treatment, at treatment termination and 3, 6- and 12-months following treatment termination.

Symptoms of depression were measured by the Patient Health Questionnaire (PHQ-9 [33];) with a maximum score of 27, where higher scores indicate more severe problems. Internal consistency in the present sample was good (α = .81). Symptoms of anxiety were measured by the Generalized Anxiety Disorder Questionnaire (GAD-7 [34];), with a maximum score of 27. Internal consistency in the present sample was excellent (α = .91).

### Baseline and post-treatment measures

Difficulties in ER were measured with the Difficulties in Emotion Regulation Scale (DERS-16 [35];), a short version of the DERS which has shown good psychometric properties among adults [36] with scores ranging from 16 to 80. Internal consistency in the present sample was excellent (α = 0.94). Psychological flexibility, a core concept in Acceptance and Commitment Therapy (ACT), was measured as an adjacent construct to ER, using the six-item Acceptance and Action Questionnaire-II (AAQ-II [37];) with a maximum score of 42, where higher scores indicate more psychological inflexibility and experiential avoidance. The instrument has shown high internal consistency (Cronbach’s α = .85) for a one-factor solution.

Gambling urges were measured with the 6-item Gambling Urge Scale (GUS [38];), scored from 0 to 3 with maximum score of 18, an instrument previously used in evaluations of CBT interventions for gambling problems with high internal consistency (α = .81) [39]*.* In addition, the Craving Experience Questionnaire for Gambling (CEQ-G [40];) measured gambling cravings in terms of frequency and intensity within the last week, with 16 items scored from 0 to 10 and a maximum score of 160; higher scores indicate stronger and/or more intense cravings. Participants were also asked to provide a brief description of their most prominent craving experience (if any) within the last week and relate intensity measures to this episode. No psychometric properties are available for the CEQ-G. Alcohol consumption was measured with Alcohol Use Disorders Identification Test (AUDIT [41];) which has shown high internal consistency (α = 0.77) in a Swedish population [42].

### Qualitative measures

Qualitative data collection consisted of a semi-structured interview on treatment acceptability and feasibility. The interview was based on the Client Satisfaction Questionnaire - 8 (CSQ-8 [43];) an 8-item questionnaire with scores of 8–32, where higher scores reflect higher satisfaction with treatment and the Treatment Acceptability Questionnaire (TAQ [44];) and asking the participants to elaborate on their answers. Two additional open-ended questions were added, prompting the participants to reflect upon possible improvements and pros and cons with the treatment. The participants were asked to give a numeric rating (closed questions) ranging from 0 to 3, a response-scale corresponding to, e.g. “poor” to “excellent” or “definitely not” to “yes, definitely” and then give a rationale or reflect upon their rating (open question). The interviewer was unknown to the participants in order to reduce socially desirable responses.

### Treatment

The treatment manual included both traditional CBT-components and additional components derived from Emotion Regulation Group Therapy (ERGT [45];). The definition of ER overlaps with third wave cognitive behavioral therapies, such as ACT, where the goal is to increase psychological flexibility and acceptance while pursuing goals in the direction of a valued life [46]. This means that some ER-related treatment components are best described as derived from ACT.

The two-hour group sessions, led by two clinical psychologists, were each organized in two parts, the first structured around an analysis of gambling behavior (AGB) and a review of participants’ homework assignments, and the second focusing on the session topic; see Table 2 for an overview of session topics. The first part of each session concerned cognitive, emotional and contextual antecedents and consequences of gambling or cravings to gamble episodes. Both contingencies from gambling sessions and successful efforts to abstain from gambling were analyzed. The homework assignments consisted of values clarification, self-observations of emotional responses and antecedents to gambling cravings as well as tracking the consequences of both gambling and alternative behaviors in valued direction. The second part of the session introduced the day’s specific topic, which this point onwards was integrated into subsequent analyses of gambling behavior. After the session topic was introduced and discussed, participants were given homework assignments together with texts covering the session topic. Each participant also received a brief individual session within the first 3 weeks of treatment aiming to clarify personal motivation and set goals for participating in treatment. One participant brought along a concerned significant other to participate in one of the sessions.

**Table 2:** An overview of group session content

(1) Introduction to session structure and homework assignments, discussion of relapses, emergency measures and introducing analysis of gambling behavior (AGB)	
(2) AGB, brief mindfulness exercise (BME), values clarification, steps in valued direction and reflecting upon consequences of gambling	
(3) AGB, BME, identification of emotions and their function in gambling and psychoeducation about gambling	
(4) AGB, BME, acceptance and problem-solving skills	
(5) AGB, BME and managing difficult emotions	
(6) AGB, BME and gambling cognitions	
(7) AGB, BME and using defusion techniques with gambling cognitions	
(8) Continuing progress, identification of obstacles discussing values and steps in valued direction, repeating components and discussing prevention of relapses	
(9) Booster session, 3 months after session 8: content review, relapse prevention, problem solving and AGB	

Four therapists in total were involved in delivering the treatment; author VM acted as therapist in all three groups, with a different co-therapist in each group. All therapists were licensed clinical psychologists with training in Cognitive Behavioral Therapy (CBT) and experience of treating patients with GD. Therapists were introduced to the treatment manual and procedure during a half-day training session.

### Data analysis

Quantitative data was managed as follows. To assess the distribution of continuous outcomes, QQ-plots and the Shapiro-Wilks test were used. First, a General Estimating Equations (GEE) model with exchangeable correlational structure was attempted, but this model may not be suitable since data would not meet the assumption of missing completely at random (MCAR). Primary and secondary outcomes were analyzed using a Generalized Linear Model (GLM) with a Cluster Bootstrap, using time as the main effect. Since treatment was delivered at the group level, bootstrapping used treatment group as an identified cluster, instead of re-sampling from the whole sample. The data were analyzed in R version 3.6.2 with the ClusterBoostrap package [47]. Analysis of treatment completers’ characteristics was based on a definition of completers as participants who had participated in five or more sessions and had completed post-treatment measures. A comparison of completers’ and non-completers’ characteristics was done using independent sample t-tests with bootstrapped confidence intervals for continuous characteristics and Chi-square tests for categorical characteristics. Timepoints were coded by the week of administration (e.g., baseline = week 0, session 1 = week 4, 3-month follow up at week 25, 6-month follow-up at week 38 and 12-month follow-up at week 64).

Qualitative interviews were recorded and transcribed verbatim, and transcripts were analyzed using theoretical Thematic Analysis (TA [48];), where the domains were pre-defined. This method was chosen to increase the relevance of the participants’ responses to the research questions. The interviews were read and re-read, and an initial coding was conducted by identifying paragraphs into a descriptive subtheme. After all interviews were coded, the codes were organized into overarching themes summarizing the sub-themes. Codes were developed by author VM, who also took part in treatment development and delivery, and all codes and sub-themes were reviewed and discussed with author AHB to achieve consensus. Illustrative quotes from each theme were selected, and each tagged with identifiers consisting of participants’ age and gender.

## Results

### Participant characteristics

The sample consisted of mainly male participants (81%) and the mean age was 36.3 (SD = 9.0) years. The sample was highly comorbid, with 81% meeting criteria for at least one additional psychiatric disorder, where depressive disorders were the most common. The average number of attended sessions among the participants was 6.3 (SD = 2.2). No significant differences were identified between completers and non-completers.

### Primary and additional outcomes

The primary and additional outcomes revealed a normal distribution, Shapiro-Wilks test G-SAS, W(20) = 0.95, *p* = .346 and the additional outcomes, GAD-7, W(20) = 0.91, *p* = .066 and PHQ-9, W(20) = 0.97 *p* = .723). The main outcomes showed a negative estimate, indicating a trend towards reduced GD-symptoms over time (β: -0.1599, 95% CI: − 0.2526 to − 0.0500). Neither depression nor anxiety symptoms changed significantly from baseline to 12-month follow-up (β: -0.0421, 95% CI − 0.0953 to 0.0203 for depression; β: -0.0297, 95% CI: - 0.0296 to 0.0367 for anxiety). No changes were observed pre- to post-treatment for difficulties in ER, nor at 12-month follow-up. See Table 3.

**Table 3:** Means and standard deviations of observed primary and secondary outcome measures

**Outcome**	**Pre-treatment**	**Post-treatment**	**3-month follow-up**	**6-month follow-up**	**12-month follow-up**	**Hedges´** ***g*** **Pre- to post- treatment** **[95% CI]**	**Hedges´** ***g*** **Pre- to 12-month follow-up [95% CI]**
G-SAS	25.8 (10.68)	16.7 (9.59)	16.1 (8.04)	13.6 (8.70)	13.7 (10.19)	0.73 [0.34, 1.14]	1.03 [0.59, 1.49]
GUS	13.4 (10.93)	5.5 (7.33)	3.8 (5.55)	2.2 (5.78)	6.6 (8.39)	0.53 [0.16, 0.91]	0.44 [0.07, 0.81]
DERS-16	41.20 (14.58)	35.1 (9.51)	31.6 (9.92)	30.2 (9.02)	36.4 (17.18)	0.05 [-0.69, 0.78]	0.10 [-0.45, 0.65]
GAD-7	16.3 (5.54)	5.7 (3.54)	6.0 (4.18)	5.2 (3.75)	5.8 (7.21)	1.69 [1.13, 2.29]	1.73 [1.16, 2.34]
PHQ-9	12.7 (5.53)	6.4 (4.73)	6.4 (4.83)	6.4 (4.91)	8.3 (6.93)	1.33 [0.84, 1.85]	0.59 [0.21, 0.98]
AAQ-II	28.4 (10.64)	19.3 (9.71)	19.7 (10.91)	19.0 (9.76)	18.7 (8.91)	0.97 [0.54, 1.42]	1.06 [0.62, 1.53]
CEQ-intensity	41.8 (18.74)	25.4 (19.08)	24.0 (19.10)	21.9 (20.67)	34.1 (21.69)	0.54 [0.17, 0.92]	0.27 [-0.08, 0.63]
CEQ-frequency	28.7 (17.19)	17.5 (16.79)	14.6 (15.39)	11.9 (17.06)	16.3 (21.12)	0.53 [0.16, 0.92]	1.34 [0.85, 1.87]
ASRS	29.7 (14.63)			20.0 (9.81)	22.2 (15.13)	0.42 [0.06, 0.79]	0.23 [-0.12, 0.59]
AUDIT	8.4 (4.15)	6.2 (3.46)	8.3 (2.89)	8.8 (4.6)	6.3 (5.10)	0.67 [0.28, 1.07]	0.69 [0.30, 1.09]

*Note.* *G-SAS* Gambling Symptoms Assessment Scale, *GUS *Gambling Urge Scale, *DERS* Difficulties in emotion regulation Scale, *GAD-7 *Generalized Anxiety Disorder Scale, *PHQ-9 *Patient Health Questionnaire, *AAQ II *Acceptance and action questionnaire,  *CEQ *Craving Experience Questionnaire, *ASRS *Adult ADHD Self Report Scale (reported Hedge´s g from pre-treatment to 6 months follow up), *AUDIT *Alcohol Use Disorder Identification Test

The mean number of symptoms according to the SCI-GD interview declined from 7.0 (1.81) at baseline to 2.1 (2.36) at follow up. Among those available for SCI-GD interview at 12- month follow-up, 4 out of 12 (33%) still qualified for a GD diagnosis.

### Time and money spent

**Fig. 2:**
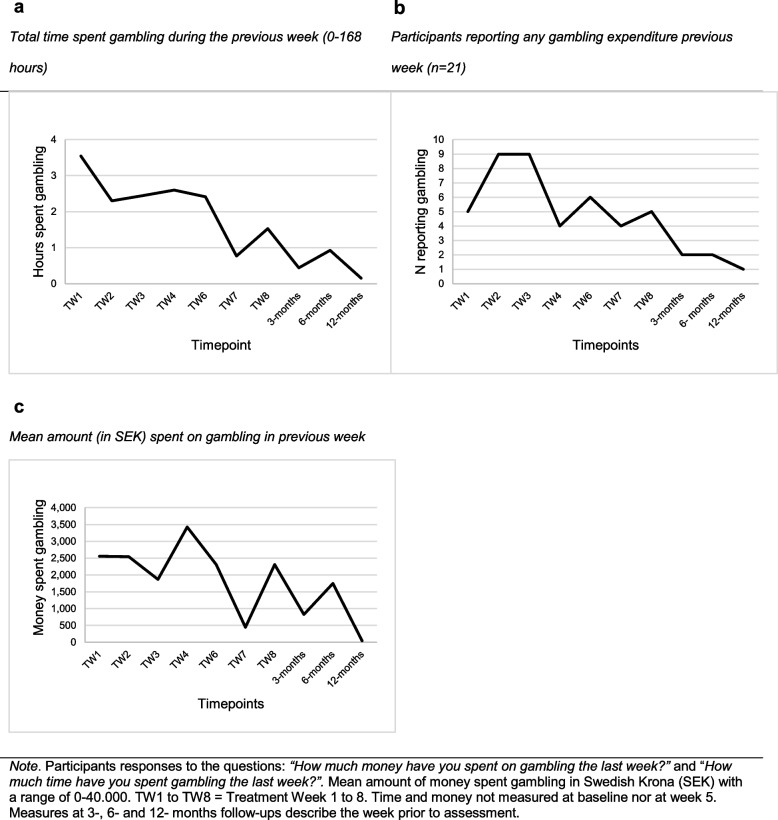
**a** Total time spent gambling during the previous week. **b** Participants reporting any gambling expenditure previous week. **c** Mean amount (in SEK) spent on gambling in previous week

Weekly measures of time and money spent gambling (Fig. 2abc) indicated a declining trend. At 12-month follow up, one participant (Fig. 2b) reported gambling expenditures during the previous week.

### Acceptability and feasibility measures

Participants’ post-treatment mean score on the CSQ-8 was 27.3 (2.74, range 24–32). Item 8 yielded no variance, meaning all would come back to the service if seeking treatment again; for items 1–7 Cronbach’s alpha was acceptable (α = .79). The TAQ score showed a mean of 32.31(2.90, range = 27–36). See Table 4.

**Table 4:** Means and standard deviations for Client Satisfaction Scale (CSQ)-8 and Treatment Acceptability Scale (TAQ) collected through telephone interview post treatment

Item	Mean (sd)	Scale
CSQ 1. How would you rate the quality of the treatment you have received?	3.3 (0.47)	1 = Poor; 4 = Excellent
CSQ 2. Did you get the kind of treatment you wanted?	3.1 (0.62)	1 = Definetely not; 4 = Definetely
CSQ 3. To what extent did the treatment meet your needs?	3.1 (0.73)	1 = None, 4 = All
CSQ 4. If a friend were in need of similar help, would you recommend the treatment to him or her?	3.7 (0.47)	1 = Definetely not; 4 = Definetely
CSQ 5. How satisfied are you with the amount of help you have received?	3.1 (0.66)	1 = Complety unsatisfied; 4 = Very satisfied
CSQ 6. Has the treatment you received helped you to deal more effectively with your problems?	3.6 (0.51)	1 = No, made it worse; 4 = Yes, to a large extent
CSQ 7. In an overall general sense, how satisfied are you with the treatment you have received?	3.3 (0.61)	1 = Completely unsatisfied; 4 = Very satisfied
CSQ 8. If you were to seek help again, would you return to the service?	4.0 (0)	1 = Definetely not; 4 = Definetely
TAQ 1. How acceptable do you find the treatment?	5.8 (1.37)	1 = Completely unacceptable; 7 = Very acceptable
TAQ 2. How ethical do you think this treatment is?	6.5 (0.76)	1 = Completely unethical; 7 = Completely ethical
TAQ 3. How effective do you think this treatment is?	5.1 (1.38)	1 = Very ineffective; 7 = Very effective
TAQ 4. How likely do you think that this treatment might have negative effects?	1.9 (1.04)	1 = Very unlikely; 7 = Very likely
TAQ 5. How knowledgeable do you think the therapist were?	6.2 (0.80)	1 = Not knowledgeable; 7 = Very knowledgeable
TAQ 6. How trustworthy do you think the therapists were?	6.9 (0.27)	1 = Very untrustworthy; 7 = Very trustworthy

### Feasibility interviews and thematic analysis

Fourteen participants took part in the post-treatment feasibility and acceptability interview. The length of the interview varied from 13 to 35 min. The results of the analysis are reported according to the themes *keys to success and treatment components*, *treatment delivery* and *potential negative effects,* with sub-themes below.

#### Keys to success and treatment components

Overall, the specific components mentioned as helpful were the analysis of gambling behavior (AGB), awareness and coping with emotions*,* being given written homework assignments, and the psychoeducational parts. The participants were divided regarding the value of the brief mindfulness exercises, where some commented them as “not helpful” and others as important. The AGB focus during the sessions was mentioned as important, in particular when the analysis involved describing emotional processes for participants who identified their gambling as a way to regulate emotional experience: “Recognizing situations and recognizing what emotion you experience, I like that it was a lot of focus on emotions.(...) I am like that, I gamble on emotions a lot.”

The texts handed out at each session was helpful as a reminder of themes covered in treatment such as raising emotional awareness and strategies to cope with difficult emotions. One participant describes: “...these parts, about coping with emotion. It was perhaps not just one session but several... it was, it was so good, well described in these texts we were given, and I still read them. They are very useful.” Another participant highlighted the importance of coping when experiencing craving to gamble: “What happens in one’s head and how to think in order to calm oneself, even if one cannot make the craving go away, but how to calm oneself in the moment, that has been the best part”.

And for some participants with a history of concealing or even lying about one’s gambling, being open and honest was an important part of the treatment.

It felt a bit melancholic to terminate everything because you have become a group, you have come close to each other, these stories, everybody has been very honest regarding everything. That is often what your problem has been about, when you had this problem that you kept things to yourself, but there you felt like you could let go and tell it like it is.

Participants with previous experiences of treatment of GD or peer support groups mentioned the importance of zero tolerance for gambling and a more punitive approach towards relapses. This was weighed against the importance of an open atmosphere amongst the group members where participants were able to speak openly about relapses.

I think people have felt that they could be honest (..) if they have gambled during previous week and it has not been judgmental in any way (..) you should be, not harsh, but in some way show that, this was not good that you have gambled, because it feels like it was more that you could come there [to the session], and then there were no consequences at all after.

General comments on keys to treatment success were that treatment facilitated “a new way of thinking”, or “gaining a different perspective”.

Other factors not specified in the treatment manual related to treatment success were the *importance of seeking treatment* in itself, *avoiding gambling situations*, taking *personal responsibility* for one’s problems, weekly *self-assessment as a reminder*, *self- determination to stop gambling* and *maintaining abstinence during treatment*.

#### Treatment delivery

The sub-themes related to treatment delivery were *dose* and *group setting*. A majority of participants expressed a need for adding treatment sessions. Some viewed the treatment as the “start of a change process” and that terminating treatment was difficult after being open about their gambling.

The fact that the treatment was delivered in a group was also a common theme, although participants were somewhat ambiguous to this format. On the one hand, there were several comments on the benefits of being in a group, such as the reduction of stigma and gaining new perspectives on one’s problem through hearing about the gambling problems of others. The need for more individual attention was mentioned and some interviewees suggested combining the group with tailored individual sessions.

#### Potential negative effects

The majority of participants did not make any statements indicating that this treatment would lead to any negative effects, such as increased symptoms or other detrimental effects nor was unethical in any way. However, issues raised as potentially bothersome included answering the same questions in weekly measures or hearing descriptions of relapses by other participants, where both could have the potential to trigger gambling urges.

If you haven’t decided to one hundred percent that ‘I will do everything in my power to stop.’ (...) If you hear the others who have gambled and their stories, I think that you yourself could experience urges...(..) it depends on where you are simply in your ‘stop-gambling-process’.

Some interviewees expressed that listening to others was not always helpful. One participant reflected on the least helpful part of the treatment for a casino-gambler to hear challenges of a someone who mainly gambled on sports:

“It was hearing the experiences of others, since all were very different and [played] different games. Of course, you could recognize oneself but still not, we all had the same basic problem, but…”. Some of the participants were struggling with negative consequences in their relationship to their partners and brought up the importance of involving concerned significant others in a more structured way, such as a devoted session for couples.

## Discussion

This study examined the feasibility and acceptability of adding emotion regulation strategies in treatment for individuals with GD and investigated within-group quantitative outcomes over 1 year. Participants rated the treatment high in satisfaction and acceptability, and highlighted the importance of treatment delivery factors such as the need for individual tailoring and prolongation of treatment. In addition, GD-symptoms declined significantly over time and time and money spent gambling showed a declining trend. However, no significant changes were noted for additional outcomes such as anxiety, depression and ER difficulties.

The treatment under investigation had a focus on emotion regulation strategies which might be more beneficial for those whose gambling serves as a way to reduce negative emotional experiences. Participant descriptions highlighted the importance of increased awareness of emotions and adopting focused strategies when experiencing craving. This is in line with research showing that individuals with GD typically experience lower emotional awareness and emotional clarity in addition to difficulties in assessing adaptive strategies [49]. Not being able to make sense of one’s emotional experiences might prevent one from making rational choices and lead to more impulsive strategies. Whether difficulties in ER precede gambling problem or vice versa is unclear, although the former is typically suggested [50]. Additionally, the *emotionally vulnerable* (EV) gambler according to the Pathways model [51] typically has a history of mood or affective disorder prior to initiating gambling.

The current trial was designed before the publication of recent studies that have described the complex relationship between ER and gambling problems. In a cross-sectional study, a somewhat paradoxical positive relationship has been found between the use of reappraisals and more gambling-related cognitive biases. Individuals with GD seem to use this strategy to justify gambling fallacies and thus neglect gambling losses [52]. In the present study participants were subject to continuous tracking of (negative) consequences through analysis of gambling sessions and the qualitative responses highlighted the importance of honesty in reporting gambling as a key to success. One might speculate that this indicates a reduction of positive re-appraisal of harmful consequences from gambling and a step towards acceptance of negative emotional states.

Gambling cravings seem furthermore to be more triggered by the lack of positive experiences, rather than the presence of negative affect. Coping with boredom and low-stimuli states seems pivotal in recovery, an idea not novel in the research field [53]. The low tolerance of boredom might be a link between emotional difficulties and gambling problems, typically manifested during initial phases of abstinence. Cross-sectional data show a mediational effect of ER difficulties between problem gambling and depression and a lack of flexibility in the use of emotional regulation strategies [15]. Accordingly, individuals with GD have been described as commonly experiencing *anhedonia* [54], a state lowering the pleasurable rewarding experience of activities.

Even though problem gambling is no longer classified as an impulse control disorder, being able to control impulses is crucial when recovering from gambling problems. Using the DERS, Marchica, Mills [15] found in a linear regression that emotion regulation difficulties accounted for approximately 4% of the variance in problem gambling, in particular the impulsivity subscale. Individuals with GD represent a heterogeneous group and a sub-group might benefit more from a stricter focus on impulse control management and others from a more cognitive approach focusing on dysfunctional gambling cognitions and a self-deceptive cognitive style, such as that presented in the Gambling Space Model (GSM [55];).

Our finding of an observed change in acceptance-related but not emotion regulation strategies is somewhat surprising, given the similarity of these concepts. One might speculate that the observed change in acceptance based on the AAQ could partly be explained by a learning effect since this measure was included in the weekly measures during treatment, whereas a measure of emotion regulation was included only at pre- and post-treatment, 3-, 6- and 12-months follow-ups.

Regarding regulation of craving, our results suggest that less *frequent* experiences of craving occurred for those remaining in treatment, yet the *intensity* of the most prominent experience remained present. For an individual striving for abstinence from gambling behavior, experiencing cravings can be troublesome and competing motives can be experienced. With increasing awareness of the overwhelming negative consequences of gambling, cravings might be associated with more stress and anxiety. Noteworthy is that the instrument we used to measure craving, the CEQ-G, has not yet been validated in clinical samples and results should be interpreted carefully. The more commonly used urge scale, GUS, showed a mean reduction of 6.8 points, close to clinically significant. A reliable urge reduction from baseline to follow-up has been suggested to be 8.57 on the GUS [56]. It must be noted that the time frame of GUS is “here and now”; i.e., dependent on current context or affective state.

The most effective format for ER-enhanced CBT is not known and is probably subject to individual preferences and possibilities of participating in treatment. Research findings are unclear as to whether group CBT-treatment is equally effective as individual treatment for problem gambling. In one study it was found that treatment outcomes of both formats were similar [57], but those in the group condition did not attain better outcomes of anxiety and self-esteem compared to the waitlist condition. Furthermore, at 6-month follow-up, 40% of those in the group treatment still met the diagnostic criteria for GD, compared to 8% receiving individual treatment. This finding was replicated by Oei, Raylu [39], where larger effect sizes could be observed among participants completing individual therapy as compared to the group format. Misreporting, underestimation and concealing of gambling behavior could be a hindering factor in a group setting. On the other hand, the group provides a potential training ground for behavior change and analysis of gambling in the presence of others, possibly lowering concealment of gambling.

The fixed number of sessions of treatment manuals can be a challenge in clinical settings. A subgroup of individual with GD might benefit from a small amount of sessions, perhaps even a single one [58] while others might need more sessions than the treatment includes, a common theme among the participants in this study.

The number of sessions needed to treat GD is unclear, but a recent meta-analysis indicated that more treatment sessions is associated with better outcomes [10]. Participants in the present study had a fairly long duration of illness and high levels of comorbid psychiatric conditions, which supports that participants’ suggestions to increase the treatment dose could be defended. Together with our finding that no changes occurred in emotion regulation strategies as measured by DERS, the qualitative responses indicating that more sessions were needed point towards insufficient treatment doses, at least for some participants.

### Strengths and limitations

This study had several strengths. First, the sample had high ecological validity, due to recruitment from a clinical population, with high rates of psychiatric comorbidity and long-term histories of gambling problems among several participants. Secondly, the mixed methods design, combining feasibility interviews and quantitative measures, yielded a more in-depth understanding of participants’ experience, particularly regarding the content and administration of the intervention in addition to changes in outcome measures. Self-ratings of satisfaction in therapy are known to have a ceiling effect, but adding interviews allowed participants to express more critical views, as interviews can be seen as promoting a more reflexive stance and deeper contemplation than questionnaires [59].

The main study limitation was the lack of a control group and not assessing the potential feasibility of randomization procedures in this context. An additional limitation was not collecting data on to what extent the participants engaged in homework assignments between sessions, as well as not asking at follow-up about participation in other treatments after termination of the study. The use of an online diary throughout treatment might have benefitted completion of homework assignment and might also have added information regarding change processes in treatment and contributed to insight regarding recovery aided by factors outside of treatment. The participants rated the treatment high in satisfaction (27.3, SD = 2.74). As a comparison, a recent study of problem gamblers who received a web-based intervention or email support and had a mean score of 25.7 and 23.5 on the CSQ-8 [60].

Individuals with GD enter treatment with a variety of problems in the domains of mental issues, health, relationships, financial and emotional difficulties. These circumstances can in themselves be a valid argument for a transdiagnostic approach in the treatment of GD. Future studies should investigate further the benefit of ER strategies in a face-to-face setting, allowing for additional sessions and individual tailoring. Also investigate the potential additive effect of ER compared to standard CBT in a randomized trial could be of value.

## Conclusions

Attendance rates, a trend toward improvement on primary outcomes and qualitative analysis of verbal responses to the CSQ and TAQ indicate that the use of emotion regulation strategies is feasible in the treatment of GD. Although adding emotion regulation strategies to GD treatment is feasible and acceptable, the treatment could benefit from individual tailoring and adding sessions.

## Data Availability

The data from this study is not publicly available since some of its content could compromise the privacy of the individual. If granted by the Ethics Review Authority, requests could be made to the corresponding author.

## Abbreviations

AAQ I: Acceptance and Action Questionnaire
ASRS: Adult ADHD Self Report Scale
AUDIT: Alcohol Use Disorder Identification Test
CEQ: Craving Experience Questionnaire
CSQ: Client Satisfaction Questionnaire
DERS: Difficulties in Emotion Regulation Scale
ER: Emotion Regulation
GAD-7: Generalized Anxiety Disorder Scale
GD: Gambling Disorder
GEE: Generalized Estimating Equations
G-SAS: Gambling Symptoms Assessment Scale
GUS: Gambling Urge Scale
PHQ-9: Patient Health Questionnaire
TA: Thematic Analysis
TAQ: Treatment Acceptability Questionnaire
