# Correction: Emotion regulation-enhanced group treatment for gambling disorder: a non-randomized pilot trial

**DOI:** 10.1186/s12888-023-05107-x

**Published:** 2023-08-23

**Authors:** Viktor Månsson, Olof Molander, Per Carlbring, Ingvar Rosendahl, Anne H. Berman

**Affiliations:** 1 Center for Psychiatry Research, Department of Clinical Neuroscience, Karolinska Institutet& Stockholm Health Care Services, Region Stockholm, Norra Stationsgatan 69, 7Tr, 113 64 Stockholm, Sweden; 2 Department of Psychology, Stockholm University, Stockholm, Sweden; 3 Department of Psychology, Uppsala University, Uppsala, Sweden

**Correction: BMC Psychiatry 22: 16 (2022)**

**https://doi.org/10.1186/s12888-021-03630-3**

Following publication of the original article [1], the authors identified an error in Table 3. The correct table is given below.

**Table 3:** Means and standard deviations of observed primary and secondary outcome measures

Outcome	Pre-treatment	Post-treatment	3-month follow-up	6-month follow-up	12-month follow-up	Hedges´ *g* Pre- to post- treatment [95% CI]	Hedges´ *g* Pre- to 12-month follow-up [95% CI]
G-SAS	25.8 (10.68)	16.7 (9.59)	16.1 (8.04)	13.6 (8.70)	13.7 (10.19)	0.73 [0.34, 1.14]	1.03 [0.59, 1.49]
GUS	13.4 (10.93)	5.5 (7.33)	3.8 (5.55)	2.2 (5.78)	6.6 (8.39)	0.53 [0.16, 0.91]	0.44 [0.07, 0.81]
DERS-16	41.20 (14.58)	35.1 (9.51)	31.6 (9.92)	30.2 (9.02)	36.4 (17.18)	0.05 [-0.69, 0.78]	0.10 [-0.45, 0.65]
GAD-7	16.3 (5.54)	5.7 (3.54)	6.0 (4.18)	5.2 (3.75)	5.8 (7.21)	1.69 [1.13, 2.29]	1.73 [1.16, 2.34]
PHQ-9	12.7 (5.53)	6.4 (4.73)	6.4 (4.83)	6.4 (4.91)	8.3 (6.93)	1.33 [0.84, 1.85]	0.59 [0.21, 0.98]
AAQ-II	28.4 (10.64)	19.3 (9.71)	19.7 (10.91)	19.0 (9.76)	18.7 (8.91)	0.97 [0.54, 1.42]	1.06 [0.62, 1.53]
CEQ-intensity	41.8 (18.74)	25.4 (19.08)	24.0 (19.10)	21.9 (20.67)	34.1 (21.69)	0.54 [0.17, 0.92]	0.27 [-0.08, 0.63]
CEQ-frequency	28.7 (17.19)	17.5 (16.79)	14.6 (15.39)	11.9 (17.06)	16.3 (21.12)	0.53 [0.16, 0.92]	1.34 [0.85, 1.87]
ASRS	29.7 (14.63)			20.0 (9.81)	22.2 (15.13)	0.42 [0.06, 0.79]	0.23 [-0.12, 0.59]
AUDIT	8.4 (4.15)	6.2 (3.46)	8.3 (2.89)	8.8 (4.6)	6.3 (5.10)	0.67 [0.28, 1.07]	0.69 [0.30, 1.09]

*Note. G-SAS* Gambling Symptoms Assessment Scale, *GUS* Gambling Urge Scale, *DERS* Difficulties in emotion regulation Scale, *GAD-7* Generalized Anxiety Disorder Scale, *PHQ-9* Patient Health Questionnaire, *AAQ II* Acceptance and action questionnaire, *CEQ* Craving Experience Questionnaire, *ASRS* Adult ADHD Self Report Scale (reported Hedge´s g from pre-treatment to 6 months follow up), *AUDIT* Alcohol Use Disorder Identification Test

The original article [1] has been corrected.
